# Autistic Traits Differently Account for Context-Based Predictions of Physical and Social Events

**DOI:** 10.3390/brainsci10070418

**Published:** 2020-07-01

**Authors:** Valentina Bianco, Alessandra Finisguerra, Sonia Betti, Giulia D’Argenio, Cosimo Urgesi

**Affiliations:** 1 Laboratory of Cognitive Neuroscience, Department of Languages and Literatures, Communication, Education and Society, University of Udine, 33100 Udine, Italy; valentina.bianco@uniud.it (V.B.); sonia.betti@studenti.unipd.it (S.B.); GIULIA.D'ARGENIO@phd.units.it (G.D.); 2 Scientific Institute, IRCCS E. Medea, Pasian di Prato, 33037 Udine, Italy; alessandra.finisguerra@lanostrafamiglia.it; 3 Department of General Psychology, University of Padova, 35131 Padova, Italy; 4 PhD Program in Neural and Cognitive Sciences, Department of Life Sciences, University of Trieste, 34128 Trieste, Italy

**Keywords:** autistic traits, autism, action observation, action prediction, context, priors

## Abstract

Autism is associated with difficulties in making predictions based on contextual cues. Here, we investigated whether the distribution of autistic traits in the general population, as measured through the Autistic Quotient (AQ), is associated with alterations of context-based predictions of social and non-social stimuli. Seventy-eight healthy participants performed a social task, requiring the prediction of the unfolding of an action as interpersonal (e.g., to give) or individual (e.g., to eat), and a non-social task, requiring the prediction of the appearance of a moving shape as a short (e.g., square) or a long (e.g., rectangle) figure. Both tasks consisted of (i) a familiarization phase, in which the association between each stimulus type and a contextual cue was manipulated with different probabilities of co-occurrence, and (ii) a testing phase, in which visual information was impoverished by early occlusion of video display, thus forcing participants to rely on previously learned context-based associations. Findings showed that the prediction of both social and non-social stimuli was facilitated when embedded in high-probability contexts. However, only the contextual modulation of non-social predictions was reduced in individuals with lower ‘Attention switching’ abilities. The results provide evidence for an association between weaker context-based expectations of non-social events and higher autistic traits.

## 1. Introduction

Autism spectrum disorder (ASD) consists of a range of neurodevelopment conditions characterized by deficits in reciprocal social behavior and communication, as well as by restrictive and repetitive behaviors and interests, which are present in the early developmental period [1]. Beside the other manifestations of this disorder, including social withdrawal and isolation [2], the presence of sensory abnormalities [3] and difficulties in mentalizing processes [4], it has been repeatedly shown that individuals with ASD struggle with making predictions or with deciding what to pay attention to on the basis of prior expectations about the sensory world [5]. The impairments in discerning the predictive relationships between events may not only compromise habituation processes [6], but they may also generate anxiety, resulting in the typical insistence of sameness usually observed in individuals with ASD [7].

This difficulty is further amplified when people are involved in social situations, where unpredictable interactions could take place. Indeed, dealing with social interactions in an effective way often requires the ability to anticipate others’ behavior, predicting their intentions from observing their movements. Movement kinematic information, however, may be ambiguous in many (if not most) social situations [8,9]. Thus, a social observer needs to integrate this sensory evidence with knowledge of past experiences aiming at the same goal or with contextual cues facilitating action prediction [10,11,12,13,14,15,16].

According to predictive coding accounts [17], the brain works as a Bayesian inference machine, merging prior expectations with current evidence to assess the probability of future outcomes as a result of rule learning. For instance, in the visual domain, prior contextual information is responsible for the elementary regularities that bias our perception of shape and color [18] as well as for the effects of perceptual learning on the processing of visual objects [19]. Moving to the framework of social cognition, studies challenging the comprehension of social behavior showed that individuals with typical development strongly rely on prior knowledge regarding the context in which actions are usually observed in order to recognize action unfolding [13,14,15,16], especially when perceptual information is scarce [20]. The predictive coding account may explain the perceptual impairments of ASD individuals in both the social and non-social domains. Indeed, an impaired learning curve may generate deficits in picking up statistical constancies in the environment, favoring routine and attention to details at the price of the broad picture (see [21] for review). Accordingly, reduced reliance on prior expectations relative to sensory inputs has been shown to lead to a plethora of abnormalities consistently observed in ASD individuals [21,22]. In a similar vein, although the ability to read the goal of an action during action observation seems to be preserved in ASD ([23], but see also [24,25] for contrasting results), there is consistent evidence that, when the outcome of the action is ambiguous, individuals with ASD exhibit impairments in using contextual priors to predict action unfolding [20,26,27,28]. Collectively, these observations highlight the difficulties of ASD individuals in integrating the available sensory evidence with previous experience when dealing with the prediction of both physical and social stimuli.

ASD has been traditionally considered as a clinical condition distinct from typical-development functioning [29]; however, there is consistent consensus in considering the disorder as the upper extreme of a constellation of impairments that may be continuously distributed in nature [30,31,32]. Along this continuum, people can, indeed, be characterized to a different extent by the presence of subclinical autistic traits. These traits can be measured by a self-report questionnaire, the Autism-Spectrum Quotient (AQ, [30]), which is widely used in research and clinical practice to measure autistic traits in the general population (see [33] for a systematic review). The AQ describes the subclinical autistic impairments across different domains related to either social behavior (i.e., Social skills and Communication) or non-social aspects of cognition (i.e., Imagination, Attention to detail, and Attention Switching).

Previous studies measuring autistic traits in the general population showed that high autistic traits (i.e., AQ scores > 21, 1 standard deviation, s.d., above the group mean) are associated with altered perceptual adaptation for social objects [34]. Furthermore, either individuals with ASD or typical-development individuals high in autistic traits (i.e., AQ scores, Mean = 22.13, s.d. = 5.74) presented a reduced sensitivity to context-based integration of sensory feedback with prior expectation [35]. These observations supported the view of a greater reliance on new information relative to prior information in ASD and high-autistic–trait individuals. A recent study [15] extended these findings to action scenarios, by investigating the association between autistic traits and motor responses during the observation of others’ actions embedded in contexts. Indeed, observing actions embedded in contexts that are congruent or incongruent with the unfolding kinematics, respectively, facilitated or inhibited discrimination performance and motor activation, as compared to observing actions embedded in an ambiguous context [14,15,16]. Notably, lower sensitivity to contextual information and higher reliance on the sensory evidence provided by kinematics were found in individuals with higher autistic traits [16], in particular in the domains of Social skills and Attention to details [16,36]. This points to an association between both social and non-social aspects of autistic traits and impairments in the integration of sensory evidence and contextual information.

Crucially, there is extensive evidence that the behavioral traits associated to social and non-social aspects of cognition may be independently distributed in the general population, pointing to different neurocognitive bases ([37] for review). However, it is still unclear how social and cognitive aspects of autistic traits might be related to the use of previous experience to predict social or non-social events. In the present study, we sought to investigate at what extent the presence of autistic traits might interfere with the ability to implicitly learn the associations between a contextual cue and a specific event (i.e., contextual priors), and to use this association in order to make predictions under perceptual uncertainty. The relation between the amount of social or cognitive autistic traits and context-based predictions were tested in a social domain pertaining the prediction of actions and in a non-social domain pertaining the anticipation of appearing objects. Specifically, concerning this latter domain, we used moving shapes as stimuli in order to manipulate arbitrary associations between a contextual cue and the moving shape, thus avoiding any previously-learned semantic associations that could affect context-based manipulations for everyday-life objects. It is noteworthy that moving shapes have been used as control stimulus for action observation in previous neuroimaging and brain stimulation studies. In particular, a study by Schubotz and co-workers [38] showed that sequential presentations of biological (i.e., action) and non-biological abstract stimuli (i.e., circles) share the recruitment of premotor regions, while triggering distinctive patterns of activations in other fronto-parietal areas. In a similar vein, a more recent study by Paracampo and co-workers [39] asked participants to predict the outcome of hand or shape movements and found that, in spite of comparable difficulty, only the first task was affected by interferential stimulation of the left primary motor cortex. In both of these studies, moving shapes were used as control stimuli for assessing the action-specific or the domain general brain involvement in predictive mechanisms. Capitalizing on these studies, here we adopted an action prediction and a shape prediction task to assess the use of prior information in driving prediction across the two domains.

In the present study, we hypothesized that, in both domains, participants should be biased towards the implicitly-learned contextual priors in order to compensate for perceptual uncertainty. Moreover, we hypothesized that the presence of autistic traits involving social aspects might relate to a reduced reliance on contextual priors to predict the unfolding of actions, while those involving non-social aspects of cognition, especially attention switching, might relate to a reduced reliance on contextual priors to predict the fate of physical events (i.e., object appearance).

## 2. Materials and Methods

### 2.1. Participants

Seventy-eight healthy young university students (49 Female, mean age = 24.12, s.d. = 6.06 years) participated in the study. We determined the sample size for testing the effects of a single predictor in the multiple regression design of our study (number of predictors = 5 AQ scales) through the G*POWER software [40]. Based on the results of a previous study on the predictive effects of the AQ subscales on action-context integration in adults with typical development [16], we estimated a medium effect size of f^2^ = 0.15 [41] and set the significance level at α = 0.05, and the desired power (1 − β) at 0.9. Participants were recruited at the University of Udine. All participants were right-handed [42] and had normal or corrected-to-normal vision. The study was approved by the local Ethics Committee (Prot. N. 47/2015/Sper, 26/05/2015 Comitato Etico Regionale Unico, Friuli Venezia Giulia, Italy) and was carried out in accordance with the ethical standards of the 1964 Declaration of Helsinki. All participants were naïve to the aims and hypothesis of the experiment and provided their written informed consent prior to the enrollment in the study. Only after the end of the whole experiment, participants were debriefed about the experimental hypothesis.

### 2.2. General Design

We used two separate prediction tasks, requiring participants to observe videos of social actions, in an action prediction task, or videos showing moving geometrical shapes, in a shape prediction task. In particular, we presented these videos in two different alternative force choice (2AFC) tasks in a paradigm including a probabilistic learning (familiarization) phase followed by a prediction (testing) phase and allowing the assessment of contextual prior in making predictions [20] when sensory evidence is scarce. Notably, the strength of priors in driving prediction is thought to be especially brought into play in 2AFC, given that top-down signals encoding conditional expectations might influence obliged decisions regarding the physical nature of sensory input [43].

A within-subject design was used. For each participant, the experiment consisted of two separate experimental sessions during which the action prediction task and the shape prediction task were administered, respectively. Each task session lasted around 20 min. The familiarization phase comprised a total of 160 trials equally divided into 4 identical familiarization blocks, while the testing phase was made of a total of 80 trials, presented in 2 identical testing blocks of 40 trials. Each testing block was administered after two familiarization blocks, allowing a few minutes’ rest between blocks. The order of the two tasks was counterbalanced across participants. At the end of the two task sessions, participants’ autistic traits were assessed by administering the AQ questionnaire (AQ, [30]).

### 2.3. Stimuli and Tasks

**Figure 1:**
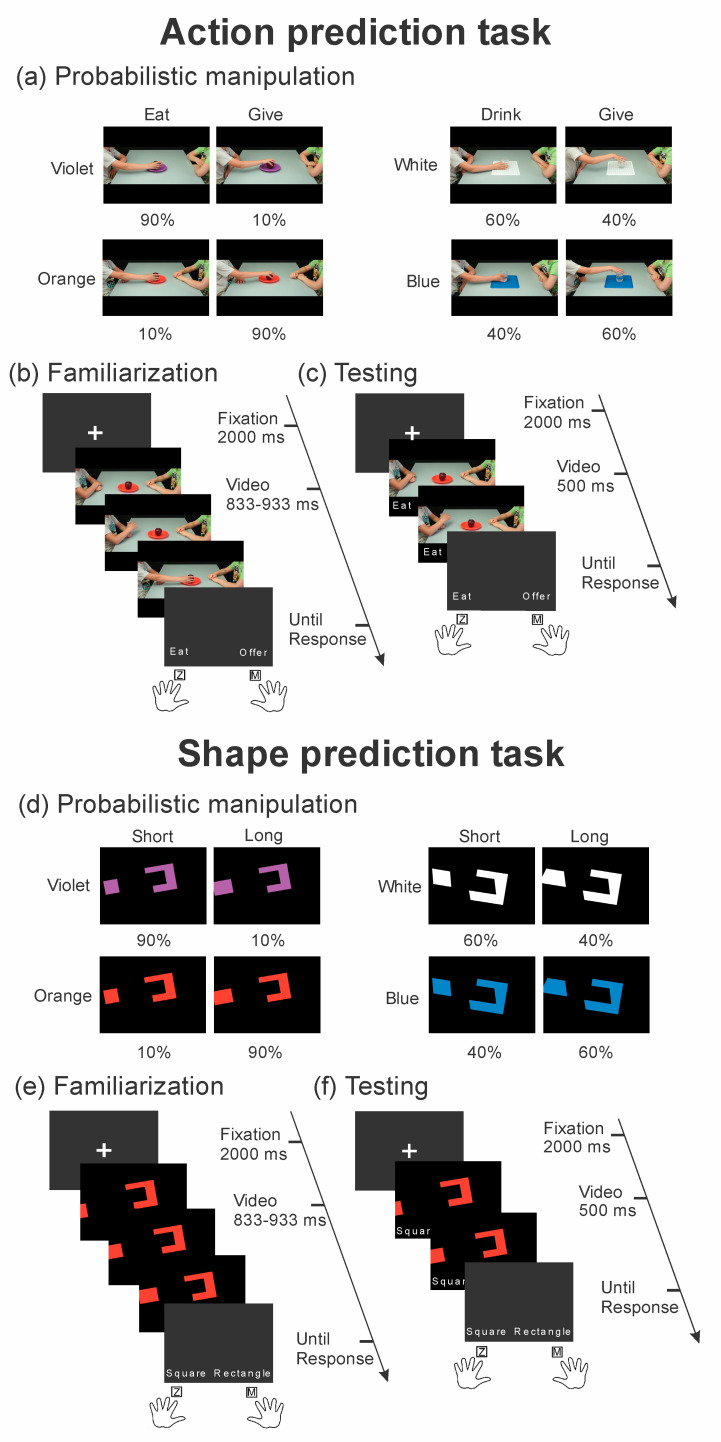
Stimuli and Experimental tasks. Action prediction task: (**a**) sketch of probabilities manipulation for the familiarization phase. Action-context associations were manipulated in terms of their probability of co-occurrence to 90%, 10%, 60%, and 40%; (**b**) familiarization phase showing videos of a child performing individual or interpersonal actions. Participants had to predict action unfolding; (**c**) testing phase showing the same videos during familiarization but of shortened duration. Participants had to predict action unfolding, as during the familiarization phase. Shape prediction task; (**d**) sketch of probabilities manipulation for the familiarization phase. Shapes-context associations were manipulated in terms of their probability of co-occurrence to 90%, 10%, 60% and 40%; (**e**) familiarization phase showing videos of moving shapes approaching the receptor shape. Participants had to predict shape identity; (**f**) testing phase showing the same videos during familiarization but of shortened duration. Participants had to predict shape identity, as during the familiarization phase.

Action Prediction Task. For the action prediction task, the same videos and paradigm proposed by Amoruso et al. [20] were used. During this task, participants watched videos showing a male child (10 years old) who was sitting in front of a peer and was grasping with his right hand an object, an apple or a glass, to perform either an individual action (to eat, to drink) or an interpersonal one (to offer). Based on the kinematics of the hand approaching the object, two possible hints could be suggested: reaching for grasping the object from its side signaled the action of moving the object toward the mouth with the individual intention of eating or drinking, while reaching for grasping the object from its top prompted the interpersonal action of offering the object. Notably, each action was performed in the presence of specific contextual cues: for actions performed upon the apple, the two possible contextual cues were a violet or an orange dish; for the actions performed with the glass, the two possible cues were a white or a blue tablecloth. In this way, stimuli consisted of a total of 8 different videos (Figure 1a).

For both phases (familiarization and testing phase) of this task, participants were asked to watch the videos and to predict the action unfolding (eat/drink versus offer). However, videos of the familiarization and testing phase dramatically differed in their length and thus in the amount of visual information provided. Indeed, videos of the familiarization phase were interrupted after the hand pre-shaping and the reaching phase, two frames before the hand contact with the object, when the amount of visual information was sufficient to distinguish the individual from the interpersonal action (Figure 1b). Differently, videos of the testing phase were interrupted during the hand pre-shaping phase, thus when the movement kinematic was still ambiguous (Figure 1c). It is important to note that, even though participants watched only the initial part of the videos, when these were originally recorded, the child was asked to perform the complete action in order to provide reliable kinematics information. For further details on video recording, please refer to [19].

Crucially, during the familiarization phase, we manipulated the probability of co-occurrence between each action and the contextual cues. More specifically, the probability of co-occurrence was set in order to have the 90%, 60%, 40% or 10% of trials representing an action-contextual cue association (namely, for 36, 24, 16, and 4 trials, respectively). For instance, an apple presented on a violet plate could be grasped 90% of the time to eat and 10% of the time to offer. In the same block, an apple presented on an orange plate could be grasped 90% of the time to offer and 10% of the time to eat. For the actions performed on the glass presented in the same block, a glass presented on a white tablecloth could be grasped 60% of the time to drink and 40% of the time to offer. Conversely, in the same block, a glass presented on a blue tablecloth was grasped 60% of the time to offer and 40% of the time to drink (Figure 1a). In this way, we sought to implicitly manipulate prior expectations regarding the action unfolding, based on the presence of these contextual cues. Importantly, the probabilistic manipulation was kept identical across the two repetitions of the familiarization phase within each participant, but we counterbalanced across participants the probability of associations between each action and a given contextual cue.

The same instructions were given during the familiarization and the testing phases. However, according to Amoruso et al. [20], in this last phase during which video duration was drastically reduced, given the perceptual uncertainty generated by the ambiguity of action kinematics, a response bias towards previously acquired contextual priors should occur. Notably, during this phase, all possible action-contextual cues associations were equally presented, thus 10 trials for each of the 8 action-contextual cue video associations were included in the whole testing phase. Before the beginning of the task, participants received information regarding the identity of the objects and demonstrations of the different possible ways of manipulating them. More specifically, we provided participants with specific examples with the original objects used in the videos (e.g., ‘this is how we grasp an apple when we want to offer it’). However, explicit information about the associations between contextual cues and actions were not provided.

#### Shape Prediction Tasks

We developed this task to compare context-based predictions in social and non-social domains. To this aim, the same logic of the action prediction task was followed in designing the task.

Video frames were created in Power Point (Microsoft Corporation, Redmond, WA, USA). They depicted colored geometric shapes appearing from the left side of the screen and moving toward the right, where a still complementary receptor figure was presented. The moving geometric shapes could be a right-angle polygon (a square or a rectangle) or an acute-angle polygon (a parallelogram or a trapezoid). The shapes of each pair of polygons looked similar on their right side, which was immediately visible at the beginning of the movement. However, with the increased visibility of the horizontal segments during the movement, the identity of the specific polygon could be detected according to the ratio between the major and minor axis of the figure, thus according to whether the horizontal segment was longer than (i.e., rectangle or trapezoid) or equal to (i.e., square or parallelogram) the visible right vertical segment. The still receptor hosted, on its left side, a concavity that was suited for binding the moving shape (Figure 1d). As in the action prediction task, we manipulated specific contextual cues based on the color of the moving shape and receptor. In particular, the square and the rectangle could be colored either in orange or in violet, while the parallelogram and the trapezoid could be colored either in white or in blue. This way, 8 different videos were created (Figure 1d). The receptor always had the same color of the moving shape, thus facilitating the salience of the color cue since the very beginning of the video.

For both the familiarization and the testing phases of this task, participants were asked to observe the videos and to predict the moving shape. Importantly, during the familiarization phase, the shapes fully appeared on the screen and thus could be easily identified (Figure 1e). Conversely, during the testing phase, videos were interrupted just one frame after the halfway appearance of the horizontal segment, thus providing minimal information about the ratio of the major-minor axis of the shape and its identity (Figure 1f). In both phases, we varied, across participants, two different response modality versions of the same task. For a first response modality (position response), which was administered to 38 participants, the left side of each complementary receptor contained two concavities, respectively, in its lower and upper parts; each concavity could host only a specific moving shape. Participants were required to report the upper or lower position of the receptor that could host the moving shape. For the second response modality (naming response), which was administered to 40 participants, the receptor presented only one concavity that could bind both possible shapes in each pair (i.e., the square or the rectangle, for one receptor, and the parallelogram or the trapezoid for the other). Participants were required to report the polygon name of the moving shape (please refer to the *Procedure and trial structure* section for further details). This way, we manipulated the contribution of the spatial and verbal ability demands that could affect the difficulty of the shape prediction task and that could be differently loaded in the two tasks.

Crucially, during the familiarization phase, we manipulated the probability of co-occurrence between each polygon and its color, using the same probability settings of the action prediction task. For instance, a violet shape could end up appearing as a square 90% of the time and as a rectangle 10% of the time. Conversely, an orange shape could end up appearing as a rectangle 90% of the time and as a square the remaining 10% of the time. In the same block, a blue shape could be a trapezoid 60% of the time and a 40% of the time, while a white shape could be a parallelogram 60% of the time a trapezoid 40% of the time (Figure 1d).

The same instructions were given in both phases. However, as in the testing phase, video duration was drastically reduced and therefore the shape identity was more ambiguous; we expected participants’ responses to be biased towards the contextual priors (i.e., shape–color associations) acquired during the familiarization phase. Notably, during this phase, all possible shape–color associations were equally presented, namely 10 trials for each of the 8 shape–color associations.

As in the action prediction task, the probabilistic manipulation was kept identical across the two repetitions of the familiarization phase within each participant, but we counterbalanced the probability of associations between each polygon and a given color across participants. Before performing the tasks, participants received information regarding the identity of all the possible geometric figures by using two-dimensional paper figures. More specifically, we provided participants with specific hints regarding the similarity of the right side but different major-minor axis ratio of the two shapes in each pair. The possible associations between color and shapes, however, were never explicitly acknowledged.

### 2.4. Procedure and Trial Structure

The same procedure and trial structure were used in the two tasks. Participants were seated in front of a computer screen at a distance of about 60 cm, with their arms positioned palm down on the keyboard. Stimuli were presented using the E-Prime software (version 2.0, Psychology Software Tools, Inc., Pittsburgh, PA, USA). Video resolution was set at 1280 × 768 pixels, with a refresh rate of 60 Hz. Trials started with the presentation of a central fixation cross (remaining on the screen for 2000 ms), which was followed by video presentation (Figure 1). Videos were presented frame-by frame at a rate of 30 Hz. In the familiarization phase, videos lasted 833–933 ms (25–28 frames), while in the testing phase videos were interrupted after 500 ms (i.e., after the initial 15 frames). For the familiarization phase, at the end of each video, a response prompt was presented at the bottom of the screen until participant’s response. It showed, respectively, at the left and right of the screen, the verbal descriptors of the two possible actions (the Italian verbs “mangiare/bere” or “offrire”; in English “to eat/drink” or “to offer”) or the position/name of the two possible concavities/polygons (the Italian adverbs “sopra” and “sotto”, in English “up” and “down”; the Italian names “quadrato” and “rettangolo”, or “trapezio” and “parallelogramma”, in English “square” and “rectangle”, or “trapezoid” and “parallelogram”). For the testing phase, the response prompt appeared at video onset and remained on the screen until the participant’s response; this way, participants could provide their response as soon as they were able to guess the correct response. Participants were asked to respond with their index fingers using the left (Z) or the right (M) keys corresponding to the left or right location of the descriptors. The position of the descriptors was counterbalanced between participants. An empty black screen was presented for 1000 ms between each consecutive trial.

### 2.5. Autistic Traits Measure

At the end of the two experimental sessions, participants completed the Italian version of the Autism-Spectrum Quotient (AQ, [30,44]), a self-report questionnaire measuring autistic traits in the general population that is widely used in research and clinical practice. The AQ consists of 50 items divided into five subscales. The Attention switching subscale measures deficits in control processes of cognition and measures the ability to switch rapidly between multiple tasks. This ability is crucial in real life scenarios, where we are challenged by a constantly changing environment and we have to adapt accordingly, frequently switching attention among multiple sources of salient events. This subscale includes items such as: “I prefer to do things the same way over and over again”; “If there is an interruption, I can switch back to what I was doing very quickly”. The Attention to Detail subscale measures the tendency to focus more on individual pieces of information at the expense of perceiving the global picture. Typical items are: “I notice patterns in things all the time”; “I usually concentrate more on the whole picture, rather than on the small details”. The Communication subscale measures deficits in the skill to properly provide and receive different kinds of information and includes items like: “I frequently find that I don’t know how to keep a conversation going”; “I find it easy to read between the lines when someone is talking to me”. The Imagination subscale covers deficits in the ability to form sensory images and experiences in the mind. Example items are: “If I try to imagine something, I find it very easy to create a picture in my mind”; “I find it difficult to imagine what it would be like to be someone else”. Finally, the Social Skills subscale concerns deficits in knowing how to act in different types of social situations and is measured with items such as: “I prefer to do things with others rather than on my own”; “I find it difficult to work out people’s intentions”. For each item, participants are required to provide one of four responses: ‘definitely agree’, ‘slightly agree’, ‘slightly disagree’, and ‘definitely disagree’. Answers are scored 1 or 0 indicating the presence or absence of autistic traits. Higher scores reflect higher autistic traits, thus greater impairment in attention switching, imagination, communication and social skills abilities, as well as a perceptual bias toward details in spite of a deficit in global processing for the Attention to Detail subscale. Individual AQ total scores range between 0 and 50, while subscale scores range between 0 and 10.

### 2.6. Data Handling

Individual AQ total and subscale scores were calculated according to standard procedures [30,44]. Independent sample *t*-test (two-tailed) was used to compare the score of male and female participants within our sample and with those reported in age-, education-, and gender-matched Italian samples reported a previous study [45].

Individual performance in the familiarization and prediction phase was expressed as d prime (d’), a bias corrected measure of sensitivity in discriminating between two categories, and as response criterion (c), which checks for the existence of a bias in providing a specific response (see [46]). For the action prediction task, individual actions identified as individual were considered ‘hits’, while interpersonal actions identified as individual were considered ‘false alarms’. For both versions of the shape prediction task, short shapes identified as short were considered ‘hits’, while long shapes identified as short were considered ‘false alarms’.

Individual d’ and c values for the familiarization phase were entered into separate 2 × 2 mixed repeated measure analysis of variance (RM-ANOVA), with Task (Action vs. Shape prediction) as within-subject variable and shape-prediction Response Modality (Position vs. Naming) as between-subject factor. For the familiarization phase, the d’ and c values were averaged across the four probability conditions due to their unequal number of trials generated by the probabilistic manipulation. For the testing phase, the d’ and c values were subjected to mixed 2X2X4 RM-ANOVAs with Task and Probability (10%, 40%, 60%, 90%) as within-subject variables and shape-prediction Response Modality as between-subject factor. Estimates of the effect size were obtained using the partial η squared (*η_p_*^2^, [41]). *Post-hoc* pairwise comparisons were carried out using the Newman–Keuls test.

Based on the results of the RM-ANOVA, we calculated, for the testing phase of each task, a facilitation index by subtracting, for each participant, the d’ value obtained in the condition of lowest probability (10%) from the average d’ values obtained in the other probabilities of association (40%, 60%, 90%). In this way, we obtained individual measures of the reliance in using contextual priors to predict the unfolding action or shape.

Then, standard multiple linear regression models were tested to assess whether the individual level of contextual modulation in the two tasks was predicted by autistic traits. Scores at the five AQ subscales (Attention switching, Attention to detail, Communication, Imagination, Social skills) were entered as independent variables, while the Action or Shape d’ Facilitation indexes were entered as dependent variables. The assumptions for multiple regression analysis were met, given the presence of linear relationships between the dependent and the independent variables, and all variables were also checked for homoscedasticity and collinearity.

All analyses were implemented in Statistica software (Version number 12, Statsoft, Tulsa, OK, USA). The alpha value for all statistical tests was set at 0.05.

## 3. Results

### 3.1. AQ Scores

Analysis of the distribution of the AQ total score revealed that it was normally distributed, with skewness of 0.41 (s.e. = 0.27) and kurtosis of −0.44 (s.e. = 0.53). The individual AQ total scores ranged between 3 and 36 (Mean = 16.4, s.d. = 7.5), spanning from low (i.e., < 13) to high (i.e., > 18) levels of autistic traits [30]. As expected [30], men (N = 30; Mean = 18.3, s.d. = 8.56) tended to have higher AQ scores than women (N = 48, Mean = 15.21, s.d. = 6.58), but the difference between the two gender groups did not reach significance (t_76_ = 1.8, *p* = 0.076). Importantly, a recent study [45] describing a sample of Italian University students of fact-based humanities, which best matched our sample for age, study-field, and gender, revealed comparable distributions of AQ scores for both men (N = 29; Mean = 17.9, s.d. = 6; t_57_ = -0.21, *p* = 0.837) and women (N = 30; Mean = 17.5, s.d. = 6.9; t_68_ = 1.41, *p* = 0.163). Table 1 describes the statistics for each AQ subscale.

### 3.2. Behavioral Results: ANOVA

For the familiarization phase, the RM-ANOVA on d’ values did not report any significant effects for Task (F_1,76_ = 0.86, *p* = 0.355), Response Modality (F_1,76_ = 3.60, *p* = 0.061) and their interaction (F_1,76_ = 0.68, *p* = 0.411). Similarly, the RM-ANOVA on c values did not report any significant effects for Task (F_1,76_ = 2.12, *p* = 0.149), Response Modality (F_1,76_ = 0.89, *p* = 0.348) and Interaction (F_1,76_ = 1.87, *p* = 0.175).

**Figure 2:**
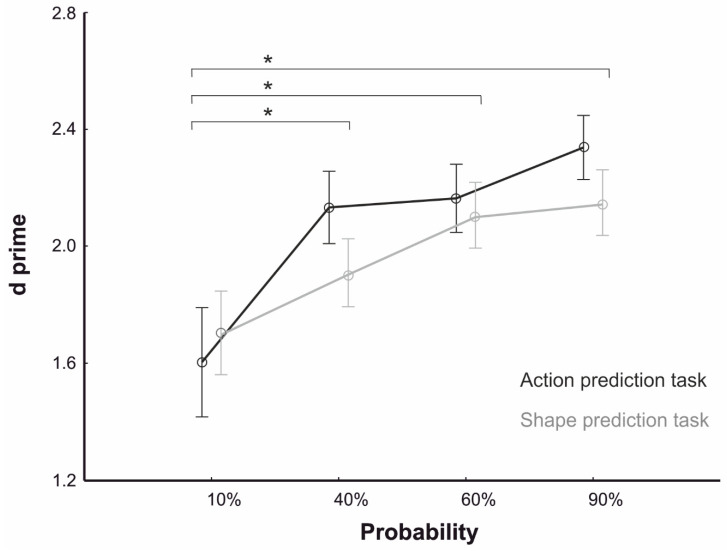
Behavioral results. Participants’ performance in predicting the action (black line) and the shape identity (grey line) for the four probability conditions (10%, 40%, 60%, 90%) expressed as d’. Data points represent group averages. Asterisks indicate significant comparisons (*p* < 0.05). Error bars represent SEM.

For the testing phase, the RM-ANOVA on d’ values yielded a main effect of Probability (F_3,228_ = 12.28, *p* < 0.001, *η*_p_^2^ = 0.13). Post-hoc comparisons revealed that d’ values were lower for the 10% (mean = 1.653; s.e. = 0.12) than the 40% (mean = 2.020; s.e. = 0.081), 60% (mean = 2.133; s.e. = 0.086) and 90% (mean = 2.242; s.e. = 0.076) conditions, thus suggesting a decreased sensitivity in target discrimination under low predictability based on the contextual cues. No significant differences were observed among the 40%, 60%, and 90% conditions (all *ps* > 0.07). Importantly, the main effect of Task (F_1,76_ = 0.49, *p* = 0.484) and the Task × Probability Interaction (F_3,228_ = 1.03, *p* = 0.376) were not significant, suggesting that the two tasks were matched for prediction difficulty and that no differences in prior modulation were present between the Action Prediction and the Shape Prediction tasks (see Figure 2). Regarding the Response Modality of the Shape Prediction Task, a main effect emerged (F_1,76_ = 16.14, *p* < 0.001, η_p_^2^ = 0.17), since we found that performing the prediction tasks was easier in Position Response (mean = 2.285; s.e. = 0.097) than in Naming Response (mean = 1.738; s.e. = 0.095). Importantly, no interaction between Response Modality and the other factors was found (Task × Response Modality: F_1,76_ = 3.77, *p* = 0.056, Probability × Response Modality: F_3,228_ = 1.11, *p* = 0.342, Task × Probability × Response Modality, F_3,228_ = 0.07 *p* = 0.974), thus ruling out a possible influence of relative difficulty of task performance due to response modality on context probability modulation.

**Figure 3:**
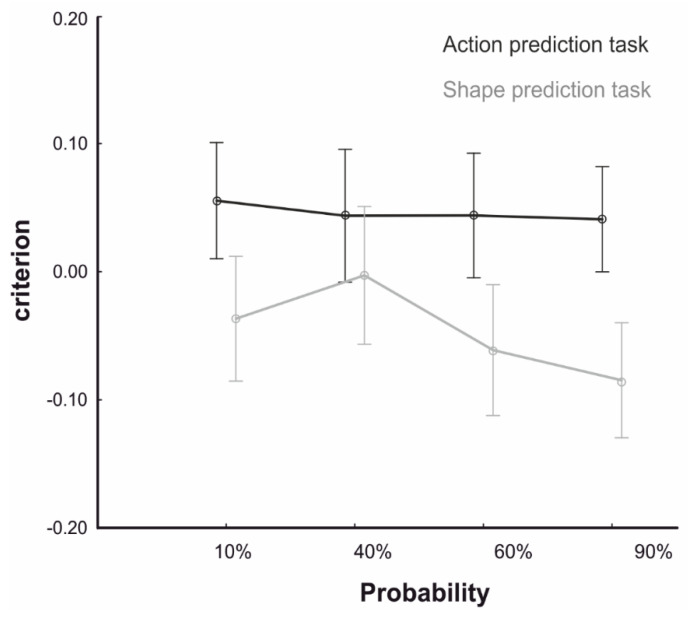
Behavioural results. Participants’ response biases in predicting the action (black line) and the shape identity (grey line) for the four probability conditions (10%, 40%, 60%, 90%) expressed as criterion (c). Data points represent group averages. Error bars represent SEM.

With respect to the response criterion, the RM-ANOVA on c values yielded a main effect of Task (F_1,76_ = 5.40, *p* = 0.023, *η*_p_^2^ =0.06), showing a negative criterion in the Shape Prediction Task (mean = −0.046; s.e. = 0.034) and a positive criterion in the Action Prediction Task (mean = 0.045; s.e. = 0.021, *p* = 0.03). This suggests that participants were differently biased in reporting one of the two outcomes in the two tasks. This, however, did not interact with Probability (F_3,228_ = 0.29, *p* = 0.833), nor was the main effect of Probability significant (F_3,228_ = 0.34, *p* = 0.799), ruling out a change in responses bias depending on the strength of the contextual prior (see Figure 3). We also found a main effect of Response Modality (F_1,76_ = 12.83, *p* = 0.008, *η_p_*^2^ = 0.14), further modulated by a significant Response Modality X Task Interaction (F_1,76_ = 20.51, *p* < 0.001 *η_p_*^2^ = 0.21). Post-hoc comparisons showed lower c values in Position Response (mean = −0.21; s.e. = 0.049) than Naming Response (mean = 0.117; s.e. = 0.047) for the Shape (*p* < 0.001), but not for the Action Prediction Task (Position response: mean = 0.062; s.e. = 0.031; Naming response: mean = 0.029; s.e. = 0.03, *p* = 0.567). This shows that the different response modalities of the two shape prediction versions led to a different bias in reporting the appearance of a long or a short shape. Importantly, no interaction with Probability was found (F_3,228_ = 0.85, *p* = 0.470), ruling out that the contextual modulation was affected by changes in responses bias across the two task versions.

### 3.3. Behavioral Results: Regression Analysis

**Figure 4:**
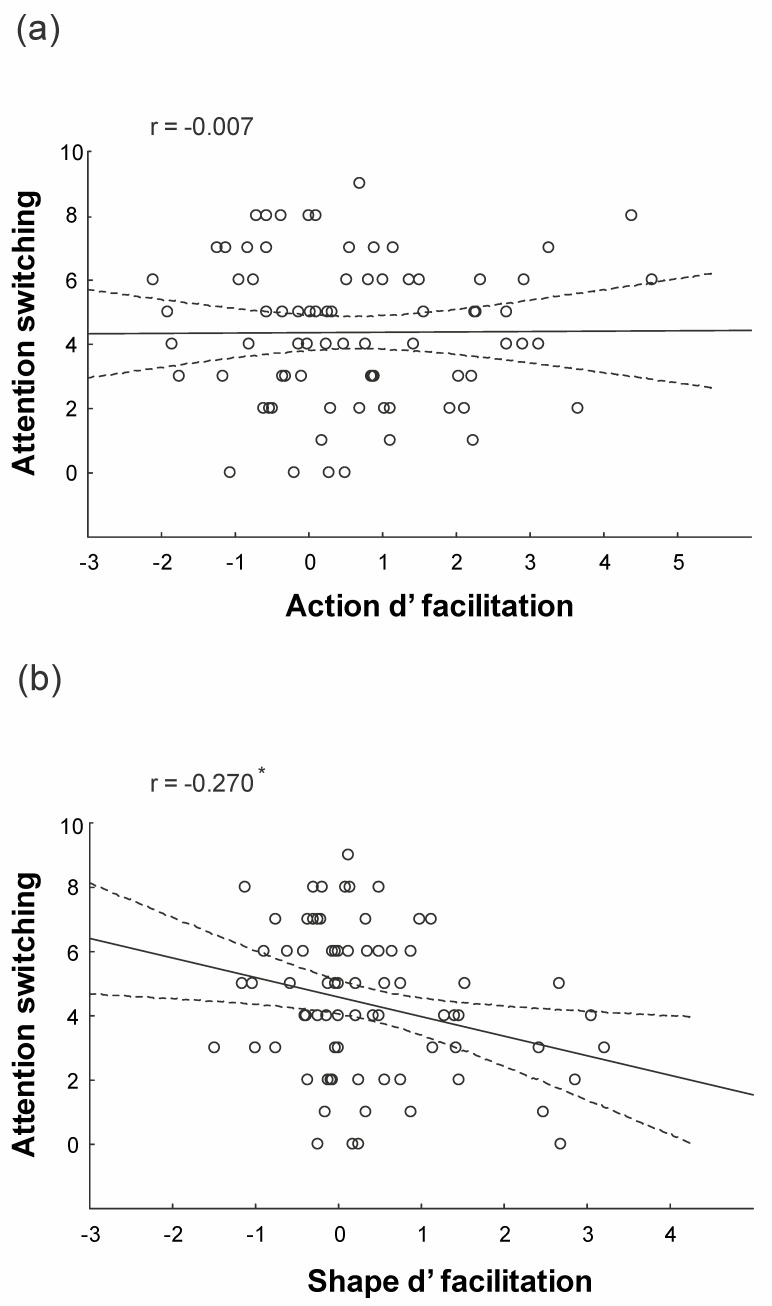
Correlational results. (**a**) lack of significant correlation between facilitation index for the action prediction task and attention switching scores; (**b**) significant negative correlation between facilitation index for the shape prediction task and attention switching scores.

Table 2 shows the summary statistics of standard multiple regression analyses conducted separately for Action d’ Facilitation and Shape d’ Facilitation indices. Multicollinearity statistics confirmed that the assumption was not violated (Tolerance > 0.4). For the Action d’ Facilitation index, none of the subscale scores was a significant predictor (whole model: adjusted *R*^2^ = −0.0461; F_5,72_ = 0.70; *p* = 0.628). Interestingly, for the Shape d’ Facilitation index, the Attention switching subscale score was a significant predictor (*p* = 0.019; whole model: adjusted *R*^2^ = 0.035; F_5,72_ = 1.561; *p* = 0.181). All the other subscale scores were not reliable predictors (all *ps* > 0.407). Thus, a higher level of cognitive autistic trait reflecting Attention Switching deficits were associated with a lower contextual prior modulation for the non-social, but not for the social task. This result was corroborated by the one-tailed Fisher transformation test, which showed that the negative correlation between Attention Switching and the Shape facilitation index (r = −0.270, Figure 4b) was significantly (*p* = 0.046) lower than the non-significant correlation between Attention Switching and the Action facilitation index (r = −0.007, Figure 4a).

## 4. Discussion

The present study tested to which extent the level of autistic traits in a general (i.e., non ASD) population could be associated with the ability to learn and use contextual priors to make predictions of social and non-social events. In line with the predictive coding account [17], we expected that contextual priors should modulate the precision of both action and shape predictions. Furthermore, in line with studies showing poor use of contextual priors for actions in ASD individuals [20] and poorer integration of action-context cues in individuals with higher autistic traits [16], we expected that social and cognitive aspects of the autistic traits should differently account for the use of contextual priors to predict the outcomes of social and physical events, respectively. In keeping with the first hypothesis, we showed that behavioral performance in predicting action and shape unfolding was significantly influenced by the strength of the contextual priors: the more the probabilities of co-occurrence of a contextual cue and a given event, the more participants were accurate in performing the tasks. This modulation was independent from the social nature of the task, since it was detected for both the action and shape prediction tasks. Moreover, it was independent from task difficulty, since we found comparable modulation for the two versions of the shape prediction task where the response modality was manipulated to load more spatial or verbal abilities. Crucially, the second hypothesis was only partially supported, since we found that cognitive (i.e., Attention Switching), but not social (i.e., Social Skills or Communication) aspects of the autistic traits accounted for the strength of contextual prior in the shape prediction task. However, we failed to find an association between either social or non-social autistic traits and use of contextual prior in the action prediction task.

### 4.1. Contextual Modulation of Social and Physical Event Prediction

The ideomotor theory [47] and forward models of action [48]) claim that the ability to predict action intention represents a crucial aspect of motor control and relies on previously learnt bidirectional associations between the motor act and its consequent sensory effects [49]). Action prediction mechanisms have been studies using different techniques. For instance, paradigms investigating the processing of anticipated action effects showed that self-generated stimuli are perceived as less intense than externally-triggered stimuli, resulting in sensory attenuation [50]. Other neurophysiological studies focused on the brain correlates of the anticipated action effects [51]. Here, we addressed action prediction from a Bayesian perspective related to the associations between action observation and contextual priors.

Using the same action prediction task of this study, a recent study [20] showed that children with typical development were able to use previously learned contextual information to successfully predict an ongoing action. In contrast, despite similar performance of children with typical development and children with ASD in discriminating fully observed actions, children with ASD did not leverage priors to predict action unfolding when visual information was ambiguous. The present study replicated the same pattern of findings in a large sample of young adult observers with typical development, thus providing evidence for consisting reliance on contextual priors in action perception across the lifespan. A recent study [52] also found that, in conditions of impoverished kinematic information available to an observer, explicit verbal information concerning the ongoing action strongly biased action prediction. In contrast, when kinematics was fully visible, the explicit verbal information had little, if no effect, with informative kinematics overriding the verbal information. The condition of fully visible kinematics was comparable to our familiarization condition, in which the unfolding action was clearly showed until one frame before the hand–object contact. Indeed, our participants could easily recognize the action outcome and used this experience in the testing phase, when kinematic information was reduced. Still, differently than in Koul et al. [52]’s study, some action-specific kinematic cues were still available during the testing phase and participants integrated this information with that provided by contextual priors, rather than overwriting kinematic evidence with the contextual prior. Thus, prediction performance was not completely biased to the contextual prior and even in the lowest probability condition participant’s sensitivity was well above 0. Of note, given that sensitivity was defined based on kinematics and not on the context, this means that kinematics was correctly identified, but performance was modulated by contextual priors. In keeping with the predictive coding view of action processing [53,54], when observing someone performing an action, our brain generates top-down expectations to explain away the perceptual kinematics. In this recursive processing, incoming kinematic evidence is continuously matched with experiential or contextual priors in order to reduce the prediction error and reach a more precise model of the perceived action. Evidence for the integration of contextual prior and kinematic information has come from neuroimaging [13] and neurophysiological [12,14,15,16,54] investigations of modulation of motor activity according to the compatibility between the observed action and the embedding context.

The present study extended the same implicit learning of stimulus-context associations to a non-social domain, namely the prediction of an appearing object based on its color. Results provided evidence of prior-related perceptual advantages in the prediction of this physical event. In line with the predicting coding theory [17], it appeared that, when observing ambiguous moving geometric shapes, our brain generates predictions to guess in a proactive way the identity of the observed stimulus, leading to a modulation of perceptual areas according to the compatibility between the object identity and the embedding context (see [55]). In this sense, future studies are needed to clarify whether the context-based modulation of motor activity during action observation is specific for actions or rather reflects a general mapping of predictability [38].

### 4.2. Attention Switching Accounts for Context-Based Prediction of Physical Events

Several studies have provided evidence of altered perception of the world in individuals with autism [56,57,58,59,60] and in typically-developing individuals with high autistic traits [61,62,63].

Three main theories have been proposed to explain the abnormalities associated with atypical visual processing in autism. The Weak Central Coherence model [64] highlights the superior focus on the local aspects of a scene at the expense of the global “bigger picture”. The Enhanced Perceptual Functioning model [65] emphasizes the enhancement in the detection of visual features. Interestingly, in a predictive coding account [65], these abnormalities may stem from an unbalance in the integration of top-down and bottom-up signals for the perception of the external world. Indeed, individuals with autism or high autistic traits may rely less on priors and more on sensorial evidence, showing a ‘hypo-priors’ mode of processing [66]. Empirical studies, however, have provided contrasting findings on whether the perceptual abnormalities of autism-like perception are due to weaker prior (e.g., [67,68]) or more precise sensorial information (e.g., [63,69]). Nevertheless, all these theories share the notion that individuals with autism-like perception do not integrate visual information in an optimal manner, thus perceiving an overwhelming sense of ‘sensory overload’ when dealing with the environment [70].

Using a task tapping the implicit learning of arbitrary shape–color associations, here we showed that high autistic traits accounted for a reduced ability to use the expectations arising from previous experience in order to discriminate among geometric shapes in situations of reduced visual information. In particular, individuals with less attention switching abilities tended not to take advantage of previously learned shape–color associations to disambiguate the appearing shape, pointing to a role of weak contextual priors in an autism-like perception of the world.

The role of a specific autistic trait linked to executive function deficits is consistent with a seminal work by Courchesne et al. [71] where authors showed an impaired ability of individuals with ASD to execute rapid attention switches during an attention shifting paradigm. Furthermore, as also demonstrated by a functional neuroimaging study using an adaptive version of the embedded figure test (EFT, [72]), participants with ASD are more dependent on perception, thus adopting a more local approach when faced with a task, compared to typically developing individuals. Notably, given the involvement of distinct cortical activations in individuals with ASD and individuals with typical development, the differences in behavioral performance seemed to be associated with different underlying neural systems, suggesting that individuals with and without autism use different cognitive strategies and engage different neural networks in performing the same task.

### 4.3. Autistic Traits Accounts for Context-Based Prediction of Actions

Several studies showed that, in individuals with ASD, although action understanding is preserved ([23] but see also [24,25] for contrasting results), impairments arise when the goal of the action has to be inferred on the basis of contextual cues [27,73], pointing to a reduced reliance on prior knowledge as the core of social interaction deficits in ASD. Therefore, we expected an association between the AQ measures of social behavior (i.e., communication, social skills) and the context-based predictions of social events. Contrary to these expectations, however, we did not find any correlation between any AQ subscales and the advantage provided by contextual cues during the performance of the action prediction task. The lack of significant results might also seem partially in contrast to previous study [16], where the authors found a negative correlation between the AQ subscales social skills and communication and neurophysiological correlates of the integration between top-down contextual expectations and movement kinematics. However, similar to the present study, the authors failed to find significant correlations between behavioral performance and AQ scores. In a similar vein, while a deficit in using contextual priors to predict action unfolding was observed in children with ASD as compared to those with typical development, the relative distribution of the deficit was not accounted for by their deficits in social perception abilities, but by their behavioral problems [20]. This might reflect that, while autistic traits may be associated with a different neural processing of social actions, this does not necessarily lead to an action prediction failure.

Otherwise, the lack of association between autistic traits and context-based predictions in individuals with either typical development or in ASD, despite the deficits shown by the last group as a whole, might reflect the dissociation between ASD and autistic traits. Consolidated views support the proposal of considering ASD as a dimensional rather than categorical disorder, with blurry and quantitative distinctions between subclinical autistic traits observed in the general population and individuals diagnosed as ASD [31,74]). Following this view, previous investigations have demonstrated the existence of a significant correlation between autistic traits and the sensory and perceptual alterations found in clinical autism [75]. Crucially, individuals with high autistic-trait scores showed similar perceptual difficulties in global processing often associated with clinical ASD than individuals with low-autistic trait scores [76]. Conversely, contrary evidence has been provided by Silverman et al. [77], who showed that autistic traits related to communication and social skills in ASD are not strongly correlated with the symptoms of stereotyped and repetitive behaviors. This is in line with the alternative view proposed by genetic studies (e.g., [78]) suggesting that ASD is best characterized as a category distinct from autistic traits [79]. Notably, to reconcile these two positions and explain contrasting findings, a categorical-dimensional hybrid model of ASD has been proposed [80,81]). This model, which has been also considered for defining the ASD diagnostic criteria in the fifth edition of the Diagnostic and Statistical Manual for Mental Disorder, has conceived the presence of either a categorical distinction between individuals with and without ASD or a dimensional representation of ASD symptoms. However, by assessing the associations between naturally varying levels of autistic traits on the ability to make experience-dependent predictions on upcoming events, without testing individuals with an ASD diagnosis, the present study does not allow establishing the extent at which clinical forms of ASD and autistic-like behaviors overlap.

## 5. Conclusions and Limitations

In conclusion, we showed that, while ASD has been associated to deficits linked to the context-based prediction of actions, the distribution of autistic traits in the general population is related to the strength of contextual priors only in the prediction of physical events. This conclusion, however, needs to be commensurate with the limitations of this study. First of all, while we powered our study to detect significant AQ prediction effects based on previous studies, it is possible that the relative uniformity of the sample, all young university students mainly enrolled in fact-based-humanities courses, might have shrunk the range of AQ variations, thus limiting predictive power. Furthermore, the unbalanced representations of male and female participants in our sample, partly related to the distribution of male and female students in the sampled university courses, might have also biased the distribution of AQ scores to lower levels of autistic traits, thus masking a possible association between action prediction and high autistic traits. Indeed, a recent study has documented an interactive effect of gender and study field on the AQ distribution in a large sample of university students [44]. In addition, using action videos depicting child models might have altered the type of action processing in our adult sample. Furthermore, we did not test here individuals with ASD and could not verify whether and how ASD and autistic traits are differently associated with context-based prediction of physical and social events. Finally, while the non-social task was designed to match the social one for difficulty and general procedure, it mapped less on everyday-life situations, thus lacking ecological validity as compared to the social task. Future studies are needed to couple the present behavioral data with neurophysiological measures and to include both behavioral and neural measures of individuals with ASD, in order to advance our understanding of the relation between object and action prediction, autistic traits, and ASD.

## Figures and Tables

**Table 1:** Distribution and collinearity indexes of the AQ subscale scores.

*AQ Subscale*	Mean	St. Dev.	Range	Skewness	Kurtosis	Tolerance
Attention Switching	4.3	2.2	0–9	−0.08	−0.75	0.507
Attention to detail	4.8	2.2	0–10	0.05	−0.45	0.847
Communication	2.0	2.0	0–8	0.87	−0.17	0.463
Imagination	2.7	1.8	0–7	0.35	−0.65	0.750
Social skills	2.3	2.2	0–9	1.03	0.30	0.413

**Table 2:** *p*-Values are marked as bold, for *p* < 0.05.

Action Facilitation Index			
*Coefficients*	β	t	*p*-Level
Attention Switching	0.051	0.316	0.752
Attention to detail	−0.073	−0.589	0.557
Communication	−0.146	−0.864	0.390
Imagination	0.219	1.648	0.103
Social skills	−0.045	−0.251	0.802
**Shape Facilitation Index**			
***Coefficients***	**β**	**t**	***p*-Level**
**Attention Switching**	**−0.375**	**−2.392**	**0.019**
Attention to detail	0.022	0.185	0.853
Communication	0.143	0.875	0.384
Imagination	−0.077	−0.597	0.552
Social skills	0.081	0.469	0.639
